# Ramadan fasting does not adversely affect neuromuscular performances and reaction times in trained karate athletes

**DOI:** 10.1186/s12970-016-0130-2

**Published:** 2016-04-19

**Authors:** Nidhal Zarrouk, Omar Hammouda, Imed Latiri, Hela Adala, Ezzedine Bouhlel, Haithem Rebai, Mohamed Dogui

**Affiliations:** Research Laboratory: “Medical Imaging Technologies” (LR 12ES06, TIM), Faculty of Medicine of Monastir, University of Monastir, Monastir, Tunisia; Research Laboratory: “Equipe de Physiologie, Biomécanique et Imagerie du Mouvement” (CeRSM, EA 2931), UFR STAPS, Université Paris Ouest Nanterre La Défense, 200 avenue de la République, 92000 Nanterre, France; Research Unit: “Exercise Physiology and Pathophysiology: from the Integrated to the Molecular Biology, Medicine and Health” (UR 12ES06), Faculty of Medicine of Sousse, University of Sousse, Sousse, Tunisia; Research Laboratory: “Sport Performance Optimization”, National Center of Medicine and Sciences in Sport (CNMSS), Tunis, Tunisia; Research Unit: “Education, Motricity, Sports and Health” (UR 15JS01), Higher Institute of Sport and Physical Education of Sfax, University of Sfax, Sfax, Tunisia

**Keywords:** Ramadan fasting, Strength, Electromyography, Cognitive performance, Reaction time, Karate

## Abstract

**Background:**

The present study aimed to investigate the concomitant effects of Ramadan intermittent fast (RIF) and muscle fatigue on neuromuscular performances and reaction times in young trained athletes.

**Methods:**

Eight karate players (17.2 ± 0.5 years) were tested on three sessions: during a control period (S1: one week before Ramadan), and during the first (S2) and the fourth week of RIF (S3). Dietary intake and anthropometric measurements were assessed before each session. During each test session, participants performed maximal voluntary isometric contractions (MVC) and a submaximal contraction at 75 % MVC until exhaustion (*T*_*lim*_) of the right elbow flexors. Surface electromyography was recorded from biceps brachii muscle during MVC and *T*_*lim*_. Simple (SRT) and choice (CRT) reaction times were evaluated at rest and just after *T*_*lim*_ in a random order.

**Results:**

The total daily energy (S2: +19.5 %, *p* < 0.05; S3: +27.4 %, *p* < 0.01) and water (S2: +26.8 %, *p* < 0.01; S3: +23.2 %, *p* < 0.05) intake were significantly increased during RIF. However, neither body mass nor body mass index was altered by RIF (*F*_(2,14)_ = 0.80, *p* = 0.47 and *F*_(2,14)_ = 0.78, *p* = 0.48, respectively). In addition, *T*_*lim*_ (*F*_(2,14)_ = 2.53, *p* = 0.12), MVC (*F*_(2,14)_ = 0.51, *p* = 0.61) and associated electrical activity (*F*_(2,14)_ = 0.13, *p* = 0.88) as well as neuromuscular efficiency (*F*_(2,14)_ = 0.27, *p* = 0.76) were maintained during RIF. Moreover, neither SRT nor CRT was affected by RIF (*F*_(2,14)_ = 1.82, *p* = 0.19 and *F*_(2,14)_ = 0.26, *p* = 0.78, respectively) or neuromuscular fatigue (*F*_(1,7)_ = 0.0002, *p* = 0.98 and *F*_(1,7)_ = 3.78, *p* = 0.09, respectively).

**Conclusions:**

The present results showed that RIF did not adversely affect the neuromuscular performances and anthropometric parameters of elite karate athletes who were undertaking their usual training schedule. In addition, neither RIF nor neuromuscular fatigue poorly affects reaction times in elite karate athletes.

## Background

Karate is currently considered as one of the most popular martial arts practiced worldwide [1]. It is divided into two competitive disciplines: kata and kumite. Kata consists of prescribed sequences of offensive and defensive techniques and movements, while kumite is a free form of sparring against an opponent. In competitions, kata performers are judged based on specific parameters: technique, rhythm, power, expressiveness of movements, and kime (i.e., short isometric muscle contractions performed at the end of a technique) [2]. The karate fight (i.e., kumite) requires high technical skills (i.e., kick and punch) with precision and high velocity to adequately execute effective attack and defense techniques [2–4]. In addition, technical performance in karate is considerably saturated by cognitive abilities and efficient attentional processes allowing more time for preparation and organization of motor behavior and ensuring quick and correct responses to visuospatial stimuli [3–7]. Therefore, reaction time, or the elapsed time between the onset of a stimulus and the initiation of a movement response [8], is crucial to achieve a high quality performance in karate techniques [2, 3]. Indeed, it has been shown that compared to novices, expert karate athletes reacted faster and/or more accurately in simple reaction time (SRT) [7], choice reaction time (CRT) [5, 6], and identical pictures test [4]. However, though it has been recently shown that reaction time was affected by some factors such as age, gender, number of stimuli and expertise in karate [9], little is known about the effects of neuromuscular fatigue on reaction time and attentional processes. In fact, the majority of the studies investigating acute effects of exercise on cognitive performance have typically incorporated aerobic (e.g., cycling or running) and resistance tasks [10]. Although aerobic and resistance exercises are common modes of exercise and considered as key determinants in achieving top karate performances, isometric exercise (i.e., kime) merits the attention since it represents the most important criterion of proper kata execution [2]. In this context, Del Percio et al. [11] showed that muscle fatigue induced by repeated isometric contractions at 50 % of maximal voluntary contraction (MVC) negatively affects visuo-spatial attentional processes in non-athletes but not in elite karate athletes. Recently, Brown and Bray [12] showed that performing isometric exercise (at 30, 50 and 70 % of MVC) until exhaustion is associated with reduced cognitive performance and that higher intensity of isometric exercise leads to greater performance impairments suggesting that exercising at high intensity levels or to exhaustion results in impaired cognitive performance.

During every day of the Ramadan month, Muslims abstain from food and fluid between dawn and sunset resulting in many effects upon individual’s physiology, biochemistry and behavior [13–15]. As major sporting calendars do not consider religious observances, competitive Muslim athletes continue to train and/or compete while undertaking the Ramadan intermittent fast (RIF). Therefore, the absence of food and fluid intake during the training as well as throughout the competition may have significant implications for physical performances. Several studies have investigated the effect of RIF upon physical performance and presented conflicting results (see for review [16]). Indeed, some authors reported significant alterations in neuromuscular performances during RIF [17, 18], while others reported no significant changes [19, 20]. However, successful performance in many sports, especially martial arts, requires not only high physical performances allowing efficient execution of motor behavior but also a high level of cognitive abilities [2, 3]. To the best of the authors’ knowledge, few studies have examined the effect of RIF on cognitive function [21, 22], and especially the combined effects of RIF and physical exercise on cognitive performances [23]. In this context, it has been reported that physical performance was not affected by RIF, while psychomotor components (i.e., recognition reaction time and total reaction time) were affected during the first week of fast in nine male resistance trained athletes [23]. Same, Roky et al. [22] observed slower CRT on the sixth day of Ramadan in ten sedentary subjects. During RIF, Tian et al. [24] found that psychomotor performance and vigilance were enhanced in the morning, however in the afternoon, verbal learning and memory were both impaired in fasted martial arts athletes.

In this way, the topic of neuromuscular fatigue and cognitive performance is of particular interest for training and competing of elite athletes involved in combat sports and especially karate. In addition, the literature reveals that the effect of RIF on neuromuscular and cognitive performances still needs to be explored. Therefore, the aim of the present study was to investigate the concomitant effects of RIF and fatigue on cognitive and neuromuscular performances in trained karate athletes undertaking training schedule during Ramadan. Given that muscle fatigue could impair cognitive performance [12], and that Ramadan fasting resulted in many effects upon individual’s physiology, biochemistry and behavior [13–15], we hypothesized that both fatigue and RIF could negatively affect reaction times and neuromuscular performances in elite karate athletes.

## Methods

### Participants

Ten right-handed male karate athletes of the Tunisian Regional Team, were volunteered to take part in this study. While eight of them (age: 17.2 ± 0.5 years; height: 175.6 ± 4.2 cm) achieved the complete experimental protocol, two athletes were excluded because of injuries during experimental period. They were at least black belt 1^st^ Dan and regularly engaged in 2 h a day, 5 days a week for at least 3 years. All the participants observed the traditional pattern of Ramadan fasting, abstaining from food and fluid from sunrise to sunset. During the study, they were regularly exercising to maintain their physical performance and undertaking their usual training sessions supervised by their coaches. Training sessions have been scheduled from 04:30 pm to 06:30 pm during control period, and after breaking the fast during RIF (i.e., from 09:30 pm to 11:30 pm [25]). The regular training sessions consisted mainly of repeated series of short-term high intensity exercises involving various basic offensive and defensive techniques (i.e., blocking, kicking, punching, displacements), sparring and katas. Some additional flexibility exercises, cardiovascular (i.e., high-intensity intermittent training) and fundamental resistance training (i.e., proprioceptive and balance exercises, core strength, and upper and lower body workout) were incorporated within these sessions. The study design is in accordance with the Declaration of Helsinki for human experimentation and was approved by the Ethics Committee of the Faculty of Medicine, University of Sousse (Tunisia). A written informed consent was obtained from the participants’ parents and participants after receiving a complete verbal description of the protocol.

### Experimental design

The study was conducted in Sousse (Tunisia) when Ramadan occurred between 22 August 2009 and 20 September 2009 (sunrise was about 05:30 am and sunset about 07:30 pm, local time). The participants were first asked to report to the laboratory 14 days before Ramadan to become familiar with the testing procedures that they would perform during the experimental sessions.

**Fig. 1:**
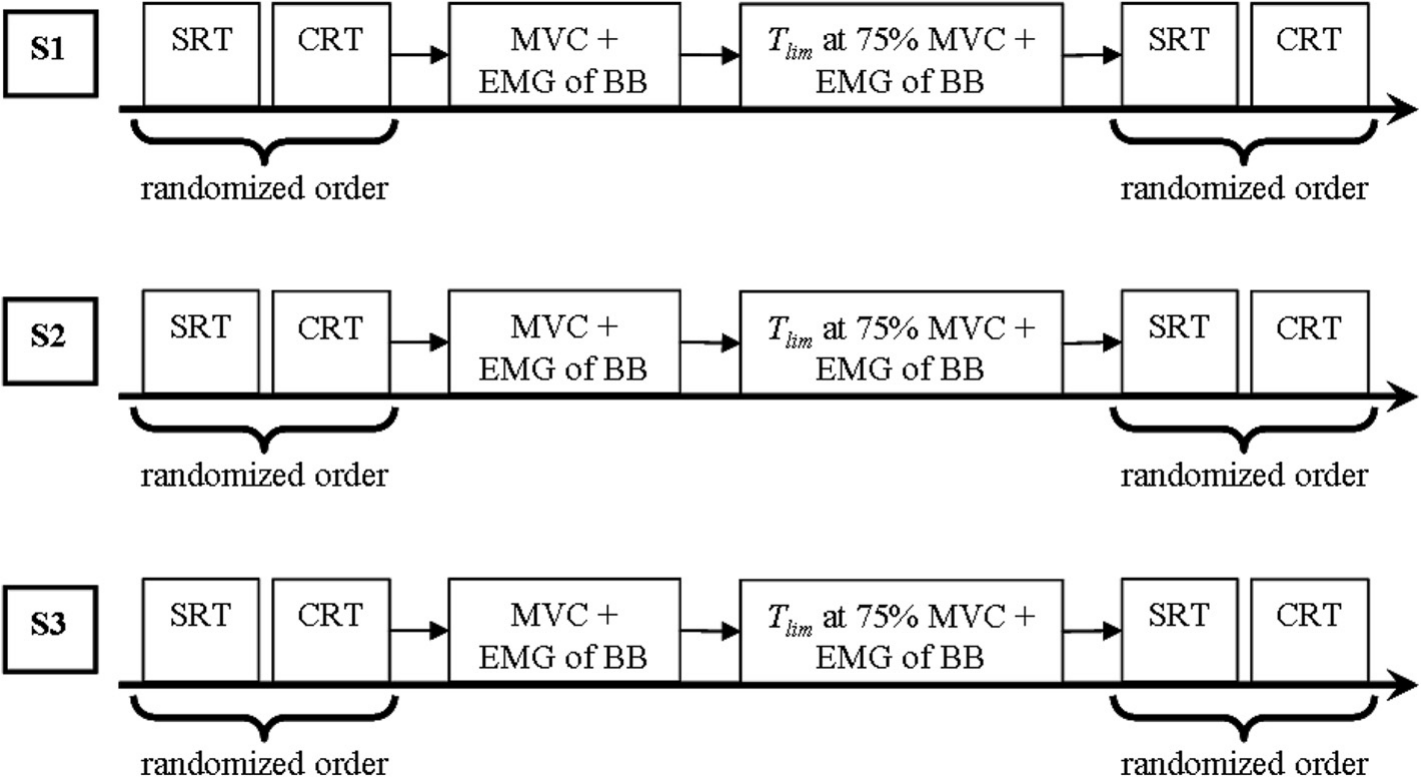
Experimental design. S1, S2 and S3: one week before Ramadan, the end of the first week of Ramadan, and the end of the last week of Ramadan, respectively; SRT: simple reaction time; CRT: choice reaction time; MVC: maximal voluntary isometric contraction of the elbow flexor; BB: biceps brachial muscle; *T*_*lim*_: isometric sub-maximal elbow flexion contraction until exhaustion

All the assessments were performed between 04:30 pm and 06:30 pm on three occasions (Fig. 1). The first study session (S1) was performed one week before Ramadan, the second (S2) was performed at the end of the first week of Ramadan, and the third (S3) was performed at the end of the last week of Ramadan. The laboratory temperature was held between 22 and 24 °C, with an average relative humidity of 56 % during the testing periods.

Dietary nutrients intake was recorded for 3 days before each session, including the last meal before the tests. This was completed by interview with the same experienced nutritionist using a 24-h recall method. Dietary records were analyzed for energy intake using Bilnut program (Nutrisoft, Cerelles, France) and values based on the food composition tables published by the Tunisian National Institute of Statistics. The height and body mass of the athletes were determined before each session using standard calibrated scale and stadiometer. The body mass index (BMI) was then calculated as body mass (in kg) divided by height (in meters squared). During each session, SRT and CRT were evaluated at rest and just after neuromuscular fatiguing exercise in a random order (Fig. 1).

### Isometric maximal voluntary contraction

During each test session, participants were seated comfortably on a Scott Bench (Panatta Sports, Apiro, MC, Italy), with the right shoulder flexed to 90°. Velcro straps secured the waist and shoulder to ensure stability and limit extraneous movements. The wrist was placed in a half-supinated position and secured by a wrist cuff using Velcro straps just below the styloid process. The wrist cuff was tightly attached to a load cell (range 0–2500 N; Globus Ergometer, Globus, Codogne, Italy) by an adjustable chain perpendicularly to the forearm. The chain was adjusted in length so that the elbow remained at 90° of flexion during the contraction (0° corresponding to full elbow extension). The position of the elbow was confirmed using an universal goniometer. The signal from the load cell was amplified using a Globus amplifier (Tesys 400, Globus, Codogne, Italy) and fed through an analog-to-digital converter (12 bit) and stored on computer with a sampling frequency of 1000 Hz.

Following a warm-up phase (sub-maximal dynamic elbow contractions) and a series of familiarization contractions (two to three sub-maximal isometric contractions followed by two to three maximal isometric contractions), participants were asked to perform three MVC of the elbow flexors. Each contraction was held for 5-s, and 3-min recovery period was provided between the attempts. All participants were given standard verbal encouragement during each MVC and visual feedback of the produced force was provided. The most forceful contraction of the three values was used for further analysis and considered the reference MVC allowing targets to be set on a visual feedback display for the subsequent endurance tasks fixed at 75 % of MVC.

### Isometric muscle endurance

Following a 10-min rest period, the muscle endurance tests were carried out using the same test apparatus described above. Participants were required to maintain an isometric sub-maximal elbow flexion (*T*_*lim*_) at 75 % MVC until exhaustion in accordance to muscle endurance task used by Bigard et al. [17] and Brown and Bray [12] (i.e., 70 % MVC). *T*_*lim*_ intensity was chosen according to the suggestion of Brown and Bray [12] who showed greater cognitive performance impairments at higher intensity of isometric contraction (30 vs. 50 and 70 % of MVC).

During the tests, the participants had a visual feedback for their contraction level on a control monitor in order to keep the output force level as close to the designated target force as possible. The endurance test ended if the level of force output appeared to have declined consistently (more than 3-s) to less than 90 % of the target force. Verbal encouragement was provided to the participants throughout the test to maintain the force level. The absolute *T*_*lim*_ was calculated as the time from the beginning of force production to the point where the above described 3-s criteria was reached.

### Electromyographic measurement and analysis

During both the maximal and sub-maximal test, electromyographic (EMG) activity was recorded from biceps brachial (BB) muscle of the right arm by using bipolar surface electrode (Delsys DE-2.1, Delsys® Inc., Boston, USA). The electrode is fitted with two silver bar contacts measuring 1 cm in length, 0.1 cm in large and with a fixed inter-electrode spacing of 1 cm. After careful preparation of the skin (shaving, abrasion, and cleaning with alcohol), surface electrode was placed parallel to muscle fibers, in accordance with the European Recommendations for Surface Electromyography [26]. The exact electrode position over the BB muscle was carefully measured for each subject and marked on the skin with a waterproof permanent marker to ensure consistent location throughout the experiment. A reference electrode was placed over the collar bone.

EMG signals were amplified (Common Mode Rejection Ratio, CMRR = 92 dB; input impedance > 10^15^ Ω; gain = 1000) using a differential amplifier (Bagnoli-4 EMG System, DelSys Inc., Boston, USA) and filtered to a bandwidth between 20 Hz and 450 Hz, using a band-pass second order Butterworth filter. The signals were analogue-to-digital converted (with 16-bit accuracy in the signal range ± 5 V; Bagnoli-4 EMG System, DelSys Inc., Boston, USA) at a sampling rate of 1000 Hz and stored in a personal computer for subsequent analysis (EMGworks 3.0 DelSys Analysis software, Boston, USA).

The EMG analysis was performed in the time and frequency domains by calculating the root mean square (RMS) and the mean power frequency (MPF). For the best MVC contraction, the EMG signal of BB muscle were analyzed over a 500 ms window centered at the highest generated force to calculate RMS amplitude. The RMS was used to calculate the neuromuscular efficiency (NME = MVC/RMS) during the MVC. For the fatigue task, RMS value and MPF of the power spectrum (512 points, Hanning window processing, Fast Fourier Transform) were calculated for consecutive 5-s windows throughout the whole fatigue task. For each participant and during each session, the changes in EMG parameters (i.e., RMS and MPF) over the entire duration of the *T*_*lim*_ were assessed by the absolute slope of the linear regression between EMG activity and time. The coefficient of determination (*R*^*2*^) was calculated for each of these relationships.

### Reaction times

The SRT and the CRT were measured using a “Superlab 4.5” program (Cedrus, San Pedro, USA). These tests measure the reaction time to visual stimuli. The subject sat 0.4 to 0.5 m in front of a computer monitor. After 10 familiarization trials, each participant performed randomly and double-blinded 20 SRT and 32 CRT tests at rest and after neuromuscular fatiguing task. The interval between the appearances of two consecutive stimuli on the monitor varied randomly between 10 and 1500 ms.

When measuring the SRT, the appearance of a white square on the monitor served as a warning signal, and the participant’s task was to press the space bar with the right hand (i.e., favored hand) as quickly as possible after the appearance of a black square. For the CRT, four white squares were presented occupying the entire monitor. The participant was then required to react as quickly as possible, pressing the key corresponding to the location of a black square (responding with a letter “A” if the black square appeared at the top left of the screen, “W” if it appeared at the bottom left, “U” if it appeared at the top right, or a letter “N” if it appeared at the bottom right).

Reaction times of less than 150 ms and greater than 800 ms were excluded from analysis to avoid any effects from either anticipation or a temporary lapse of concentration.

### Statistical analyses

All data are presented as means ± standard deviation (SD) and were analyzed using Statistica for Windows software (version 6.0, StatSoft, Inc, Tulsa, OK). The reproducibility of SRT, CRT, MVC and *T*_*lim*_ measurements was assessed by calculating the intra-class correlation coefficient (ICC) and the standard error of measurement (SEM) between the familiarization session and S1. Once the assumption of normality was confirmed using the Shapiro-Wilk *W*-test, parametric tests were performed. For all EMG variables, MVC, *T*_*lim*_, anthropometric measures and dietary data, one-way analysis of variance (ANOVA) with repeated measures was used to detect significant differences between the three sessions. For the SRT and CRT data, two-way (Sessions × pre/post exercise) ANOVA with repeated measures was used. When appropriate, the least-significant difference (LSD) post-hoc test was used for multiple pair-wise comparisons. Statistical significance was accepted at *p* < 0.05.

## Results

The reproducibility of SRT (ICC = 0.92; SEM = 11.58 ms), CRT (ICC = 0.88; SEM = 10.26 ms), MVC (ICC = 0.98; SEM = 34.60 N) and *T*_*lim*_ (ICC = 0.91; SEM = 4.40 s) was high.

Compared to the control values (i.e., S1), total daily energy intake was significantly increased during RIF (S2: +19.5 %, *p* < 0.05; S3: +27.4 %, *p* < 0.01; Table 1). As shown in Table 1, the diet pattern used by our participants during Ramadan showed a significantly greater estimated daily total fat content during the first (+34.9 %, *p* < 0.01) and the last (+46.0 %, *p* < 0.001) week of Ramadan compared to the usual diet (i.e., S1). However, there was no significant difference in the intake of protein over the whole period of the investigation. In addition, although the fractional contribution of carbohydrate to the daily diet was significantly higher before than during Ramadan (S2: −11.1 %, *p* < 0.05; S3: −10.9 %, *p* < 0.05; Table 1), no significant difference of the dietary carbohydrate content in g was found across the three sessions. Estimated total daily water intake from ingested food and fluids was significantly increased during Ramadan (S2: +26.8 %, *p* < 0.01; S3: +23.2 %, *p* < 0.05; Table 2) compared with before Ramadan (i.e., S1).

**Table 1:** Mean values (± SD) of daily dietary intake before Ramadan (S1), in the first week of Ramadan (S2), and in the fourth week of Ramadan (S3)

	S1	S2	S3
Energy (MJ/d)	13.3 ± 2.2	15.9 ± 2.1^*^	16.9 ± 2.0^††^
Energy (Kcal/d)	3173.3 ± 531.1	3774.7 ± 527.2^*^	4042.1 ± 488.9^††^
Protein (g/d)	108.0 ± 36.7	123.6 ± 21.3	128.0 ± 22.7
Protein (% of energy)	11.9 ± 2.4	12.1 ± 2.1	11.8 ± 1.4
Carbohydrates (g/d)	439.6 ± 78.3	471.1 ± 110.4	495.3 ± 66.9
Carbohydrates (% of energy)	54.1 ± 7.0	48.1 ± 5.5^*^	48.3 ± 4.7^†^
Total fat (g/d)	126.8 ± 36.4	171.2 ± 20.2^**^	185.2 ± 32.2^†††^
Total fat (% of energy)	34.0 ± 5.8	39.8 ± 5.6^**^	40.0 ± 5.6^††^
Fluid intake (l)	1.8 ± 0.6	2.2 ± 0.6^**^	2.2 ± 0.6^†^

^*, **^: significant differences between S2 and S1 at *p* < 0.05 and *p* < 0.01, respectively

^†, ††, †††^: significant differences between S3 and S1 at *p* < 0.05, *p* < 0.01 and *p* < 0.001, respectively

**Table 2:** Mean values (± SD) of the anthropometric characteristics, maximal voluntary isometric contraction (MVC), root mean square (RMS) of the EMG signal and neuromuscular efficiency of the biceps brachial muscle during MVC, and absolute endurance time at 75 % MVC (*T*_*lim*_) of elbow flexion obtained from the three sessions: before Ramadan (S1), in the first week of Ramadan (S2), and in the fourth week of Ramadan (S3)

	S1	S2	S3
Body mass (kg)	62.1 ± 7.4	61.8 ± 7.2	61.8 ± 7.1
Body mass index (kg/m^2^)	20.1 ± 2.2	20.0 ± 2.1	20.0 ± 2.1
MVC (N)	907.7 ± 230.1	878.4 ± 240.1	870.2 ± 245.9
RMS (mV)	0.7 ± 0.2	0.7 ± 0.3	0.6 ± 0.3
NME	1.4 ± 0.5	1.5 ± 0.5	1.6 ± 0.5
*T*_*lim*_ (s)	35.3 ± 16.7	42.5 ± 22.3	29.5 ± 13.2

No difference was observed among the three different sessions

As shown in Table 2, neither body mass nor BMI was altered by Ramadan fasting (*F*_(2,14)_ = 0.80, *p* = 0.47 and *F*_(2,14)_ = 0.78, *p* = 0.48, respectively; Table 2).

The ANOVA indicated that there was no significant effect of session for MVC (*F*_(2,14)_ = 0.51, *p* = 0.61; Table 2). The level of RMS activity of the BB during the MVC was also not statistically different between sessions (*F*_(2,14)_ = 0.13, *p* = 0.88; Table 2). Moreover, the NME of the elbow flexor remained similar throughout the experiment (*F*_(2,14)_ = 0.27, *p* = 0.76; Table 2).

Our data also indicate that the *T*_*lim*_ during the elbow flexion was not statistically different between sessions (*F*_(2,14)_ = 2.53, *p* = 0.12; Table 2). Table 3 gives an overview of the EMG results during the *T*_*lim*_. For all tests, a significant linear regression was found between EMG parameters (i.e., RMS or MPF) and time. More precisely, we found a positive linear regression for RMS, indicating an increase over time, and a negative linear regression for MPF, indicating a decrease over time. The one-way ANOVA indicated that the slopes coefficients of the RMS (*F*_(2,14)_ = 0.71, *p* = 0.51) and MPF (*F*_(2,14)_ = 2.73, *p* = 0.09) values did not differ between the three sessions.

**Table 3:** Analysis of the relative changes in the values of the root mean square (RMS) and the mean power frequency (MPF) of the EMG signal of biceps brachial muscle across the isometric muscle endurance task tested before Ramadan (S1), in the first week of Ramadan (S2), and in the fourth week of Ramadan (S3)

	RMS						MPF					
	S1		S2		S3		S1		S2		S3	
	R^2^	slope	R^2^	slope	R^2^	slope	R^2^	slope	R^2^	slope	R^2^	slope
Mean	0.665	0.014	0.521	0.009	0.539	0.012	0.952	−1.190	0.873	−0.933	0.939	−1.057
SD	0.278	0.008	0.335	0.008	0.327	0.007	0.071	0.680	0.095	0.623	0.066	0.586

Significant linear regression was found between EMG parameters (i.e., RMS and MPF) and time

Slope: slope of the linear regression between EMG parameters and time

*SD* standard deviation

**Fig. 2:**
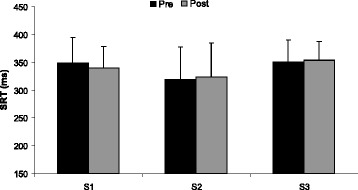
Mean values (± SD) of the simple reaction time (SRT) evaluated at rest (Pre) and after (Post) the isometric muscle endurance task before Ramadan (S1), in the first week of Ramadan (S2), and in the fourth week of Ramadan (S3)

**Fig. 3:**
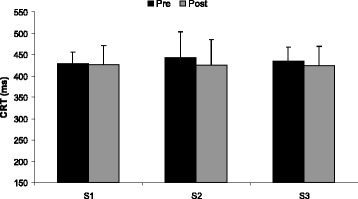
Mean values (± SD) of the choice reaction time (CRT) evaluated at rest (Pre) and after (Post) the isometric muscle endurance task before Ramadan (S1), in the first week of Ramadan (S2), and in the fourth week of Ramadan (S3)

The two-way ANOVA showed that the isometric sub-maximal elbow flexion had no effect on SRT (*F*_(1,7)_ = 0.0002, *p* = 0.98; Fig. 2) or CRT (*F*_(1,7)_ = 3.78, *p* = 0.09; Fig. 3) during any of the three sessions. In addition, there was no significant effect of RIF on SRT (*F*_(2,14)_ = 1.82, *p* = 0.19; Fig. 2) or CRT (*F*_(2,14)_ = 0.26, *p* = 0.78; Fig. 3) both at rest and after the isometric muscle endurance task without any significant interaction Sessions × pre/post exercise (SRT: *F*_(2,14)_ = 1.14, *p* = 0.35; CRT: *F*_(2,14)_ = 0.48, *p* = 0.63).

## Discussion

The aim of the present study was to examine the effect of RIF and fatigue on cognitive and neuromuscular performances in trained karate athletes who were undertaking training sessions during Ramadan. The present results showed that RIF did not adversely affect neuromuscular performances and anthropometric parameters. In addition, neither RIF nor neuromuscular fatigue poorly affects reaction times in elite karate athletes.

Similarly to previous studies [27, 28], the present findings showed that estimated total daily energy intake was significantly increased during Ramadan. Yet, other studies have reported a significant decrease [29, 30], or no significant effect [31–34] of RIF on daily energy intake. Obviously, these discrepancies could probably be related to differences in nutritional customs and habits, social and geographical environment of the country where the studies were conducted and the seasonal occurrence of the Ramadan month. In addition, differences in the characteristics of the participants such as sex, age, fitness and individual physical activity may contribute to the inconsistency of the findings across the different studies [29, 33]. The present findings corroborate the common local belief that Muslims tend to overcompensate in terms of food intake during Ramadan fasting. The dietary intake data indicated a larger increase of total fat during Ramadan despite the reduction of meal frequency. In fact, it has been reported that the diet traditionally eaten during Ramadan, in most Muslim countries, tends to be richer in calories and higher in fats, protein and sugars than the normal diet during the other months [13, 30, 33]. Of note, the mean intake composition of carbohydrate and protein throughout the study were within normal values when compared to the recommended dietary allowances (RDA), which are between 45–65 % and 10–35 %, respectively [35]. However, the mean intake composition of fat was higher during RIF (39.8 ± 5.6 % in the 1^st^ week and 40.0 ± 5.6 % in the 4^th^ week) when compared to the RDA values which are between 20 and 35 %. Despite the fact that the total fat intake was significantly higher during Ramadan than the control period, body mass and BMI did not significantly change. This observance could be due to a potential heavier training loads during RIF, and consequently higher energy expenditure. Thus, it is possible that the athletes increased their energy intake during RIF to meet the fuel requirements of higher training loads and promote optimal recovery [25]. Unfortunately, neither training load nor energy expenditure of the participants were monitored. Consequently, the present study is not able to provide a precise answer to this suggestion. Another possible explanation that needs to be considered is that the invariability of the body mass could be attributable to a possible increase in the utilization of the ingested fat during RIF. Indeed, Chaouachi et al. [33] reported that energy intake of young elite judo athletes remained constant during Ramadan but observed significant losses in body mass and body fat. The possible increase in fat oxidation during the fasting month is a probable adaptation that is due to the raised concentrations of circulating lipids and low liver glycogen levels that normally occur following several hours of fasting [29, 31, 33].

Otherwise, the present study results show that the maximal force generating capacity (i.e., MVC) and associated EMG amplitude (i.e., RMS) were not different between sessions. In addition, the NME, estimated via the MVC/RMS ratio, was unchanged during Ramadan suggesting that both muscle recruitment and contractility were not altered by the RIF during short-duration maximal skeletal muscle force. In this context, Racinais et al. [19] reported similar results in 11 moderately active Muslim males of various origins living in Qatar. These authors showed that knee extensors and flexors MVC, associated EMG and NME were maintained during Ramadan [19]. Moreover, Waterhouse et al. [20] showed that maximal handgrip strengths of both dominant and non-dominant hands were not different between control days and Ramadan. Furthermore, previous findings on changes of single maximal isometric strength [36] and explosive force [36–40] demonstrated similar observations. In contrast, other studies have shown significant impairments in short-term muscular performances (e.g., MVC, squat jump, countermovement jump, etc.) during RIF [17, 18, 34]. For instance, significant decrements in elbow flexion and knee extension MVC ranged from 10 to 15 % during Ramadan in comparison with control values were observed in fighter pilots [17]. Same, Brisswalter et al. [18] demonstrated that RIF led to an impairment in knee extensors MVC (−3.75 %) associated with decreased median frequency (−5.6 %) and RMS (−18 %) of the EMG signal from the vastus lateralis muscle. In the other hand, some studies have shown a reduction in both hand-grip strength and 30-s maximal isometric performance associated with a ~5 % body mass loss following a 7-day restricted energy and fluid intake in national-level judo players [41, 42]. Therefore, while the studies of Filaire et al. [41] and Degoutte et al. [42] were conducted with food and fluid restrictions, our results demonstrated higher estimated total daily energy and water intake during RIF which could defend against muscular performance decrements. In fact, it has been suggested that the alteration in energy substrate and metabolism, concomitant with acute hypohydration caused by the RIF could result in a reduced physical capacity to exercise [13, 43]. Otherwise, the present results did not show any session’s effect on *T*_*lim*_ at 75 % MVC and the manifestations of corresponding EMG parameters over time. It is well known that during submaximal exercise, as performed in the present study (i.e., *T*_*lim*_ at 75 % MVC), neuromuscular fatigue induces a decrease in action potential muscle conduction velocity, an increase in motor units synchronization and force loss which can be compensated by recruitment of additional motor units [44]. Consequently, these phenomena may induce an increase in EMG amplitude [45] and a decrease in the MPF of the Power Spectrum Density Function [46], while force or power output remains constant. In accordance with the literature [47, 48], the present findings showed significant positive and negative linear relationships between *T*_*lim*_ and both RMS and MPF, respectively. Notably all the participants of the present study took part in regular training sessions including resistance exercise training during Ramadan. Therefore, we speculate that due to the level of physical condition and habitual physical training, athletes were able to maintain their neuromuscular performances during the month of fast. Similar observations in subjects undertaking usual sports activities and training schedule throughout Ramadan have been reported [32, 39, 40]. Indeed, it has been shown that RIF had no adverse effect on moderate exercise in physically active subjects [43], on anaerobic performance in power athletes [32], on aerobic (i.e., Multistage Fitness Test) and anaerobic (i.e., Squat Jump, Counter Movement Jump, and maximal 30 m sprint) performances in elite judo athletes [39], on vertical jump and balance performance in female taekwondo players [40] and on speed, power, agility, passing and dribbling skills in young soccer players [34, 38]. In contrast, other studies have observed a negative effect of RIF on physical performances in competitive professional athletes [18, 34, 37, 49]. From a soccer perspective, Meckel et al. [34] reported a significant decrease in endurance and jumping performances concomitant to a reduced training load when energy intake and sleep duration were unchanged. In addition, Zerguini et al. [37] found significant reductions in agility, dribbling speed and endurance performance in professional soccer players with reduced training during RIF. While these two last studies observed reduced training load, most of the studies have reported that physical performance can be maintained during RIF once training duration, intensity, and loads are maintained compared with the pre-Ramadan period [32, 39]. It is interesting to note that both groups of soccer players in the studies of Zerguini et al. [37] and Meckel et al. [34] were free living, whereas in the study of Kirkendall et al. [38] the players stayed in a residential training camp which could provide a more physically rigorous, rigid, and healthier lifestyle than when free living. In more similar tasks to those used in the present study, Bigard et al. [17] demonstrated that muscular endurance time evaluated in elbow flexors and knee extensors at both 35 and 70 % of MVC in 11 fighter pilots were lower at the end of Ramadan in comparison with the control period (−28 and −22 %, respectively). In addition, Chaouachi et al. [39] showed significant reduction in the 30-s repeated jump test performance and an increased perception of fatigue at the end of RIF. These discrepancies in the literature might partly be related to a potential effect of mood and motivation level of the subjects and the influences of changes in sleep patterns and calorie intake [37]. Interestingly, it has also been suggested that changes in sleep habit may also indirectly impair psychomotor performance and cognitive function [15, 21, 22, 50] via changes in mental alertness, motivation, coordination and mood during RIF [13–15, 22, 51]. The present study results’ demonstrated that both reaction times (i.e., SRT and CRT) were unchanged. Unfortunately, neither sleep pattern nor mood and motivation were monitored in the present study. However, we can suggest that the karate athletes have not been suffered from a lack of sleep during Ramadan, as they were in a residential school which could provide a healthier lifestyle than when free living. In fact, Roky et al. [14] demonstrated that subjective alertness, evaluated by a visual analogue scale, decreased at 09:00 and 16:00, and increased at 23:00 during Ramadan. These authors concluded that sleep loss and reduced energy intake were responsible. The role of decreased food intake was supported by observation of improved mood in the evening after fasting had ended [14]. It has been reported that both fluid and food (particularly carbohydrate intake) deprivation, may adversely affect physical and cognitive abilities [52]. However, the present results show that the total daily water and energy intake were increased during RIF, while the dietary carbohydrate content (in g) was unchanged throughout the study. This probably reflects the experience of athletes and coaches, who ensured a compensatory increase of fluid and energy intake during the hours of darkness, when drinking and eating were permitted [25]. These findings may explain, at least in part, the unaltered cognitive performances (i.e., SRT and CRT) in the present study. Consistent with our findings, Gutiérrez et al. [53] demonstrated that perception-reaction time (simple and discriminant) and hand grip strength were unchanged after one day and three days of fasting in eight sportsmen. Regarding RIF, previous studies that have assessed psychomotor performance have reported conflicting results. In fact, while some studies have reported no adverse effects of RIF on psychomotor performance in Muslim athletes [23, 24], other studies have demonstrated that some indicators of psychomotor performance, such as critical flicker fusion [21], daytime alertness [22], irritability [50], memory [51], functional attention [54], continuous attention and reaction time [22, 55] were impaired by RIF in sedentary subjects. It could be argued that fasted athletes have a greater mental and stronger motivation to perform cognitive tasks compared to sedentary subjects [52]. Particularly, Tian et al. [24] examined various aspects of cerebral function in martial arts athletes. These authors found that psychomotor performance and vigilance were enhanced at 9:00, but verbal learning and memory were impaired at 16:00 during RIF, when blood glucose levels were presumably reduced. Lotfi et al. [23] demonstrated that critical flicker fusion and motor reaction time were unchanged during Ramadan observance in nine male resistance athletes, although the recognition reaction time and the total reaction time were impaired only at the beginning and not at the end of Ramadan. Similarly, impairments of critical flicker fusion [21] and choice reaction time [22] have been reported only in the first week of Ramadan suggesting a possible adaptation to intermittent fasting.

Furthermore, the present findings show that the muscle endurance task had no effect on reaction times. In fact, it has been reported that cognitive performance may be maintained, enhanced or impaired depending on the time when it is measured, the physical fitness level of the subjects, the type of cognitive task selected, and the type of exercise that is performed [56]. Regarding muscular exercise, Kroll [57] found no effect of purely muscular fatigue on reaction time. More recently, Del Percio et al. [11] demonstrated that tiredness and muscle fatigue after repeated isometric muscle contractions at 50 % MVC did not affect visuo-spatial attentional processes of elite karate athletes. These authors speculated that brain circuits of reflexive attention are poorly sensitive to the effects of tiredness and muscular fatigue in elite karate athletes, so that they can effectively react to unexpected kicks and/or punches even during the final part of the match characterized by high muscular fatigue [11].

The current study has some limitations that need to be addressed. First, the number of recruited volunteers is relatively low without a control group. Nevertheless, obtaining such a naturally non-fasting group in Muslim countries is somewhat difficult. Second, the present study lacks some details and measurements that would be able to strengthen the interpretation such as the training load monitoring and measurement of the energy expenditure of the athletes. Finally, some parameters (e.g., motivation, alertness, sleep quantity and quality) and factors (e.g., circadian rhythm and time-of-day effect) that we did not assess have been demonstrated to affect physical and cognitive performances during RIF. Therefore, upcoming investigations should take these parameters and factors into consideration.

## Conclusions

The present findings did not show any adverse effect of RIF on cognitive and neuromuscular performances in trained karate athletes undertaking training sessions during Ramadan. However, these results need to be confirmed for longer testing procedures (e.g., isometric muscle endurance at 50 or 25 % MVC) which could be more sensitive to intermittent fasting. In addition, further research is needed to assess the effects of RIF on reaction time using more specific stimuli and motor responses with dynamic displays of karate athletes performing offensive and defensive actions [5, 6].

## Abbreviations

ANOVA: analysis of variance
BB: Biceps brachii
BMI: body mass index
CRT: choice reaction time
EMG: electromyography
ICC: intra-class correlation coefficient
MPF: mean power frequency
MVC: maximal voluntary contraction
NME: neuromuscular efficiency
RDA: recommended dietary allowances
RIF: Ramadan intermittent fast
RMS: root mean square
S1: first session
S2: second session
S3: third session
SEM: standard error of measurement
SRT: simple reaction time
*T*_*lim*_: submaximal isometric contraction at 75 % MVC
